# p63 orchestrates serine and one carbon metabolism enzymes expression in head and neck cancer

**DOI:** 10.1186/s13062-023-00426-1

**Published:** 2023-11-09

**Authors:** Angela Cappello, Giulia Tosetti, Artem Smirnov, Carlo Ganini, Xue Yang, Yufang Shi, Ying Wang, Gerry Melino, Francesca Bernassola, Eleonora Candi

**Affiliations:** 1 Department of Experimental Medicine, University of Rome “Tor Vergata”, 00133 Rome, Italy; 2 Interdisciplinary Department of Medicine, University of Bari “Aldo Moro”, 70121 Bari, Italy; 3 Istituto Dermopatico dell’Immacolata, IDI-IRCCS, 00167 Rome, Italy; 4 Division of Medical Oncology, A.O.U. Policlinico di Bari, 70124 Bari, Italy; 5 Department of Tumor Immunology and Gene Therapy Center, Third Affiliated Hospital of Naval Medical University, Shanghai, 200438 China; 6 National Center for Liver Cancer, Shanghai, 201805 China; 7 The Third Affiliated Hospital of Soochow University and State Key Laboratory of Radiation Medicine and Protection, Institute for Translational Medicine, Soochow University, Suzhou, China; 8 CAS Key Laboratory of Tissue Microenvironment and Tumor, Shanghai Institute of Nutrition and Health, University of Chinese Academy of Sciences, Chinese Academy of Sciences, Shanghai, China

**Keywords:** Head and neck cancer, p53 family, p63, Serine, One carbon metabolism

## Abstract

**Background:**

Head and neck squamous cell carcinoma (HNSCC) is characterized by high proliferation and limited differentiation. The altered expression of the p53 family members, and specifically of p63, represents a pivotal event in the pathogenesis of HNSCC. Physiologically, p63 affects metabolism through the direct transactivation of the enzyme hexokinase 2, and subsequently controls the proliferation of epithelial cells; nonetheless, its role in cancer metabolism is still largely unclear. The high energetic demand of cancer and the consequent needs of a metabolic reshape, also involve the serine and glycine catabolic and anabolic pathways, including the one carbon metabolism (OCM), to produce energetic compounds (purines) and to maintain cellular homeostasis (glutathione and S-adenosylmethionine).

**Results:**

The involvement in serine/glycine starvation by other p53 family members has been reported, including HNSCC. Here, we show that in HNSCC p63 controls the expression of the enzymes regulating the serine biosynthesis and one carbon metabolism. p63 binds the promoter region of genes involved in the serine biosynthesis as well as in the one carbon metabolism. p63 silencing in a HNSCC cell line affects the mRNA and protein levels of these selected enzymes. Moreover, the higher expression of *TP63* and its target enzymes, negatively impacts on the overall survival of HNSCC patients.

**Conclusion:**

These data indicate a direct role of p63 in the metabolic regulation of HNSCC with significant clinical effects.

**Supplementary Information:**

The online version contains supplementary material available at 10.1186/s13062-023-00426-1.

## Background

Head and neck squamous cell carcinoma (HNSCC) is the sixth malignancy in the world by incidence [1, 2] with a significant mortality rate [3]. HNSCC represents a heterogeneous group of tumors with a communal origin from the epithelial cells lining in the oral cavity, the oropharynx, the larynx, or the hypopharynx [4, 5]. Among known risk factors, tobacco and alcohol have a synergistic effect in HNSCC onset, but also human papilloma virus and/or Epstein–Barr virus infection can play a recognized role in its occurrence [6, 7]. The viral or environmental link has a direct effect on the clinical outcome. Surgery and chemo-radiotherapy, primary treatments for HNSCC, critically impact on patients’ quality of life and tumor relapse is invariably very common [8–10]. Surgical removal and the combination of radiotherapy with chemotherapy are the first line therapeutic approaches against this type of cancer as well as in other squamous malignancies such as the esophageal carcinoma [11–14]. Although advances in therapy based on a better understanding of HNSCC pathogenesis, as the association of Cetuximab (anti EGFR monoclonal antibody) to standard chemotherapy, treatments remain, at date, not very effective and only 40–50% of HNSCC patients will survive 5 years after the diagnosis [15, 16]. From a molecular point of view, the amplification or deregulated expression of the genomic locus encoding for one of the members of the p53 family [17, 18], p63, is a frequent event in HNSCC [19], beyond the contribution of p53 itself [20–23], whose genomic alteration is present in 70% of HNSCC cases [24]. As well described elsewhere, the involvement of p53 in tumour suppression [25–27] is widely recognized [28], impinging on translation [29, 30], cell death [31, 32], epigenetic regulation [33–35] or metabolism [36–39]. p63 is specifically expressed in epithelia and regulates cellular proliferation and stem cells capacity [40–42]. An alteration of the expression of a amino-shorter isoform of p63 (ΔΝp63), codified by the second promoter, seems to play a pivotal role in HNSCC; indeed, recent studies indicate its involvement in aberrated cell survival, renewal, and senescence suppression [43–45]. Moreover, in normal epithelia ΔΝp63 is involved, together with the catalytic subunits of SWI/SNF complex (Brg1 or BRM), in maintaining a cell-type specific open chromatin to control the enhancer landscape for normal cell differentiation [45–47]. By contrast, the association between ΔΝp63 and another component of the SWI/SNF complex, ACTL6A, is responsible of the reduction of chromatin accessibility and of the alteration of gene transcription profiling typical of HNSCC [48]. In normal epithelial cells, i.d. keratinocytes, ΔΝp63 controls cellular metabolism through direct transactivation of the first enzyme of glycolysis hexokinase 2 (HK2) [49]. This evidence suggests that ΔΝp63 might be able to control cellular energy production to sustain cell proliferation [42, 49]. Cancer cells undergo a metabolic adaptation to maintain a high rate of cellular proliferation, where glucose and the amino acid glutamine are the major metabolic sources to maintain glycolysis and Krebs cycle [50–52]. The high energetic demand of cancer cells involves the anabolic pathway of serine biosynthesis where the enzyme phosphoglycerate dehydrogenase (PHGDH) converts 3-phospho- glycerate generated from glycolysis into the precursor 3-phosphohydroxypyruvate; subsequently the enzymes phosphoserine aminotransferase 1 (PSAT1) and phosphoserine phosphatase (PSPH) convert the 3-phosphohydroxypyruvate into serine [53]. Distinct studies demonstrate that PHGDH upregulation and serine biosynthesis can play a role in sustaining cancer cell growth and transformation [54, 55]. This biosynthetic pathway, known as “serine de novo biosynthesis”, refuels Krebs cycle through PSAT1 converting glutamate to α-ketoglutarate, an anaplerotic intermediate of the cycle. In general, serine synthesis represents the major source of intermediate of Krebs cycle (approximately half of the anaplerotic flux). The amino acid serine, produced by this reaction, becomes the substrate of other two enzymes, the hydroxymethyltransferases SHMT1 and SHMT2, localized in the cytosol and mitochondria, respectively [53]. The reversible conversion of tetrahydrofolate (THF) and serine to methylenetetrahydrofolate (me-THF) and glycine catalyzed by SHMTs enzymes, represents the major source of hydroxy-methyl groups (–CH3) required for glutathione (GSH), purines and S-adenosylmethionine biosynthesis (methyl groups donor for DNA/histone methylation) [56]. All together these metabolic reactions are known as one carbon metabolism (OCM), fundamental for cell energy production and homeostasis maintenance [57, 58]. In human keratinocytes the extracellular availability of serine and glycine is essential for normal cell proliferation and the intracellular inhibition of SHMTs enzymes, responsible for serine and THF conversion into glycine and 5,10 methylene-THF ameliorates psoriatic skin hyperproliferation and inflammation [59]. In addition, the modulation of the activity of SHMT2 and methylenetetrahydrofolate dehydrogenase (NADP+ dependent) 2 (MTHFD2) can affect cutaneous squamous cell carcinoma proliferation [60]. In the recent years the study of cancer metabolism, that comprises tumor and immune cell reprogramming, extracellular and intracellular metabolites availabilities for cellular transformation, migration and invasion, and the metabolic response to chemotherapy, represents a fundamental point to completely understand tumor cell transformation and/ or resistance to therapy [61–64] and to better evaluate some of the molecular alterations that are linked to HNSCC pathogenesis [65, 66].

Despite the alteration of the serine biosynthetic pathway and OCM has been shown in many human cancers, its role in HNSCC has not been yet described.

## Results

### p63, serine and OCM enzymes are altered and differentially expressed in HNSCC

**Fig. 1:**
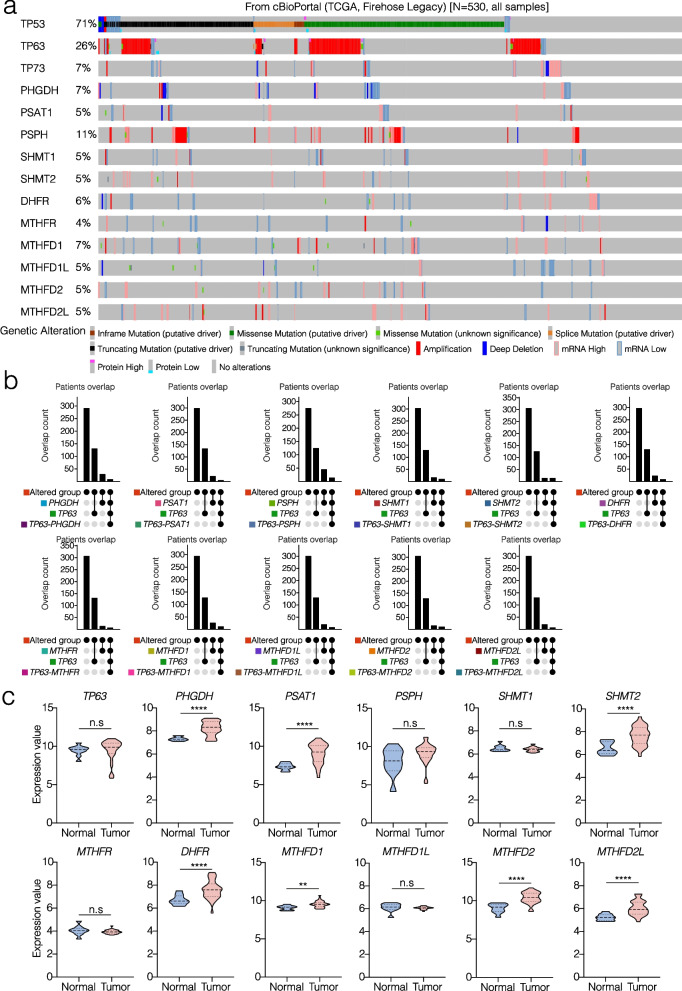
Serine and one carbon metabolism enzymes are overexpressed and deregulated in HNSCC patients. **a** OncoPrint profiling of TP53, TP63, TP73, serine de novo and one carbon metabolism (OCM) enzymes alterations in HNSCC, analyzed from the TCGA Firehose Legacy dataset on the cBioportal platform. **b** Patients’ overlap analysis of expression alterations in the OCM and serine de novo genes with TP63 in HNSCC dataset analyzed on cBioportal. **c** Expression analysis of TP63 and serine de novo and OCM enzymes using a publicly available dataset of HNSCC (GSE12452). The p-value has obtained using Student’s t test with Welch’s correction. Values were considered significant with *p* value < 0.05 (*n.s*. = not significant)

The p53 family members (p53, p63 and p73) are strongly altered at the genomic level in a cohort of HNSCC human samples analyzed with the OncoPrint profiling from the TCGA Firehose Legacy dataset through the cBioportal platform (71%, 26% and 7% genomic and/or transcriptomic alterations, respectively). Additionally, the serine de novo biosynthesis and OCM enzymes show various alterations at a genomic and transcriptional level. Among all the enzymes analyzed in the same cohort of patients from the TCGA, *PSPH* represents the most altered gene (Fig. 1a), frequently amplified. Moreover, patients’ overlap analysis of expression alterations in the OCM and serine de novo genes with *TP63* alterations in the same HNSCC dataset analyzed on cBioportal, shows a greater number of patients altered in the *TP63-PSPH* group compared to the other overlaps (Fig. 1b). The expression analysis of *TP63* and serine de novo and OCM enzymes using a publicly available dataset of HNSCC (GSE12452) shows that these genes are differentially up regulated in HNSCC samples compared to the normal control (Fig. 1c). In addition, single cell RNA sequencing revels that there is an expression gradient starting from non-lesional samples (NL), leukoplakia samples (LP) to positive lymph nodes samples (LN) and primary cancer samples (CA) (Additional file 1: Figure S1a). Altogether, this evidence suggests that OCM and serine de novo enzymes, together with p63, could be involved in HNSCC pathogenesis.

### p63 directly binds serine de novo metabolism and OCM genes promoter regions

**Fig. 2:**
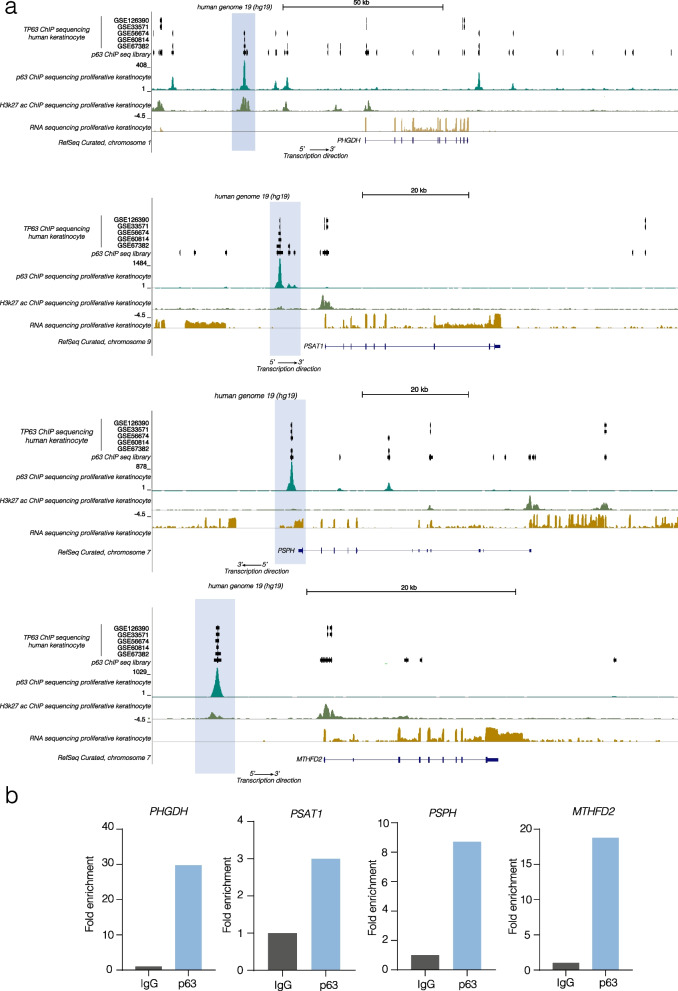
p63 directly binds promoter regions of genes involved in serine metabolism and OCM. **a** Chromatin Immunoprecipitation sequencing (ChIP seq) of p63 performed on human primary keratinocytes and HaCaT cells, available on Geo Dataset (GSE126390, GSE33571, GSE56674, GSE60814, GSE67382, GSE59827), visualized on Genome Browser (https://genome.ucsc.edu/); **b** ChIP experiment on FaDu using anti-p63 antibody to evaluate p63 binding on the promoters of *PHGDH*, *PSAT1*, *PSPH* and *MTHFD2* genes. One representative experiment is shown

To better understand if there is a mechanistic link between the epithelial master regulator p63 and serine de novo biosynthesis enzymes, publicly available chromatin immunoprecipitation sequencing (ChIP seq) of p63 performed on human primary keratinocytes and HaCaT cells (GSE126390, GSE33571, GSE56674, GSE60814, GSE67382, GSE59827) were visualized and analyzed on Genome Browser (https://genome.ucsc.edu/) (Fig. 2a). The direct binding of p63 to the promoter regions of these genes is indicated as black bands and/or peaks corresponding to H3K27ac area of open chromatin; these areas are highlighted in light blue and represent the chromatin regions where p63 can bind *PHGDH, PSAT1, PSPH* and *MTHFD2* promoters. To confirm this bioinformatic evidence, a chromatin Immunoprecipitation for p63 in the same genomic regions was performed on FaDu cells (Fig. 2b). As shown by p63 fold enrichment respect to the IgG control, we demonstrate the physical binding of this transcription factor on *PHGDH, PSAT1, PSPH* and *MTHFD2* promoters. Additionally, these enzymes result to be more expressed in single cell RNA seq of primary and metastatic cancer compared to the normal cells (Additional file 1: Figure S2a–c).

Taken together these results show that there is a direct transcriptional link between p63 and serine de novo biosynthesis enzymes and OCM enzymes.

### p63 modulates serine de novo metabolism and OCM expression levels and their molecular signature impacts on patients’ overall survival

**Fig. 3:**
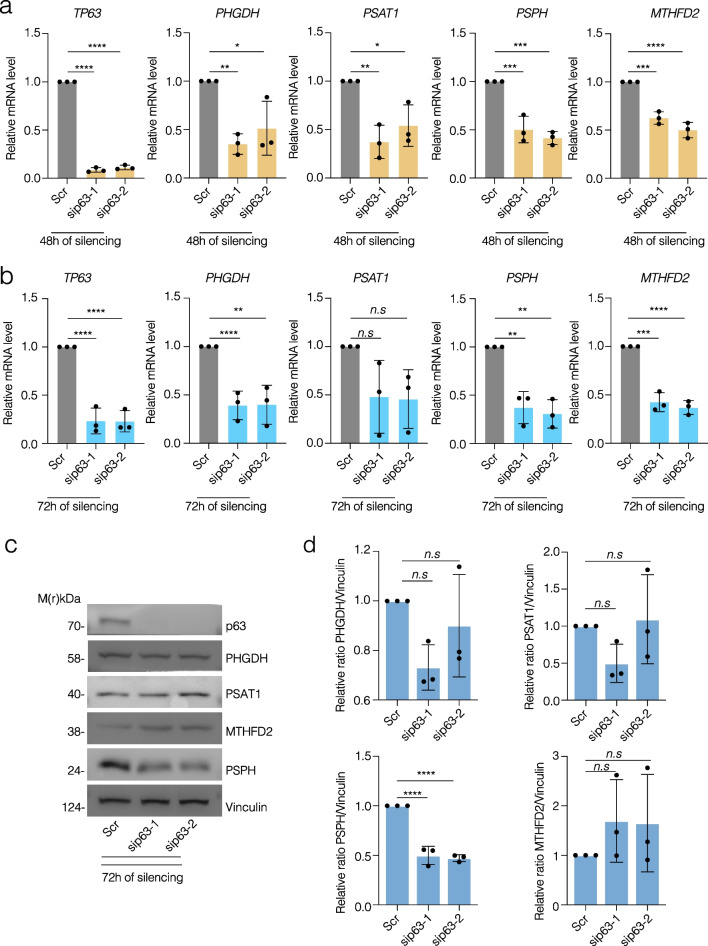
p63 silencing decreases the expression of phosposerine phosphatase enzyme (PSPH) in FaDu. **a** Relative mRNA levels of genes involved in de novo serine biosynthesis (*PHGDH, PSAT1 and PSPH*) and OCM (*MTHFD2*) after p63 silencing for 48 h; **b** relative mRNA levels of *PHGDH, PSAT1 and PSPH* and *MTHFD2* after p63 silencing for 72 h; **c** Western blot analysis of PHGDH, PSAT1, PSPH and MTHFD2 after 72 h of p63 silencing. **d** Relative ratio of PHGDH, PSAT1, PSPH and MTHFD2 after p63 silencing for 72 h, normalized protein levels. One representative experiment of 3 is shown. The p-value was obtained using ordinary one-way analysis of variance (ANOVA). Values were considered significant when *p* value < 0.05 (*n.s*. = not significant)

After demonstrating that p63 can directly bind the promoter regions of serine de novo and OCM enzymes, we performed a correlation analysis between p63 protein expression and *PHGDH, PSAT1, PSPH* and *MTHFD2* mRNA expression levels in HNSCC (Additional file 1: Figure S3a). To this end, we analyzed the mRNA expression level of *PHGDH, PSAT1, PSPH* and *MTHFD2* after p63 depletion in FaDu and HaCaT cell lines and we found a significant reduction of their expression under silencing conditions (Additional file 1: Figure S3b–c). Moreover, we performed p63 silencing with 2 different siRNA in FaDu cells showing that there is a significant reduction of *PHGDH, PSAT1, PSPH* and *MTHFD2* mRNA level at 48 h (Fig. 3a) and a significant reduction of *PHGDH, PSPH* and *MTHFD2* and a tendence to decrease for *PSAT1* at 72 h (Fig. 3b). In addition, after 72 h of silencing we also observe a significant reduction of PSPH protein level (Fig. 3c, d).

**Fig. 4:**
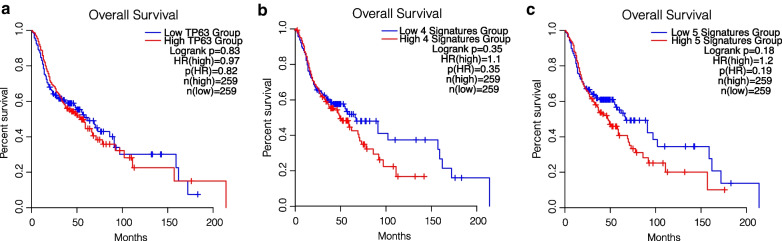
p63 and OCM mRNA molecular signature impacts on HNSCC overall survival. **a** Overall Survival analysis of HNSCC based on mRNA expression; in the first panel, patients are divided between high and low *TP63* expression, in the second panel between high and low expression of the OCM gene signature (*PHGDH*, *PSAT1*, *PSPH* and *MTHFD2*), in the last panel, between high and low expression of the TP63&OCM gene signature (*TP63, PHGDH*, *PSAT1*, *PSPH* and *MTHFD2*); the *p* value was obtained using the log-rank test. Values were considered significant when the *p* value < 0.05 (*n.s*. = not significant)

**Fig. 5:**
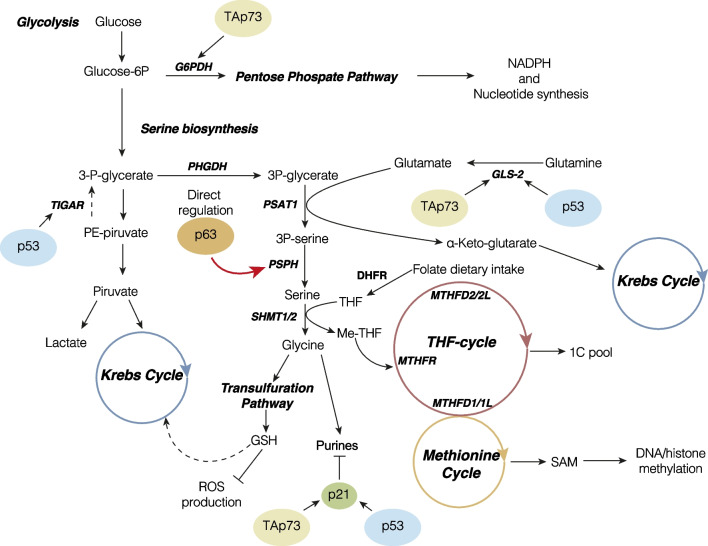
p63 controls serine de novo biosynthesis by transcriptionally regulation of PSPH. Schematic representation summarizing the biochemical pathways in which serine and OCM enzymes are involved, highlighting the transcriptional regulation by the p53 family members and, specifically, p63 regulation on PSPH gene

Despite the strong number of alterations in HNSCC, the high expression of *TP63* alone and the high expression of *PHGDH, PSAT1, PSPH* and *MTHFD2* (four-genes signature) are not sufficient to impact on HNSCC patients’ overall survival, but the high expression of *TP63, PHGDH, PSAT1, PSPH* and *MTHFD2* (five-genes signatures) seems to be more impacting on patients’ overall survival (Fig. 4a–c). All together these data confirm the importance of p63 direct control of serine biosynthesis and OCM enzymes expression in HNSCC pathogenesis which might be summarized in Fig. 5.

## Discussion

### p63-driven metabolic changes in HNSCC

Various cancers form distinct anatomical origin fall into the category of HNSCC. All these neoplasms have a common feature that is the origin from a squamous epithelium form the upper respiratory tract. HNSCC are largely caused by exogenous insults to the epithelium such as cigarette smoke and alcohol abuse. A distinct subset of HNSCC show a clear link with viral infections, specifically from human papilloma virus or Epstein-Barr virus. Cancers belonging to this group are often diagnosed at a local stage, which can be quite advanced [67], and are primarily treated with two approaches: surgery, if the mass is accessible (which is not the case of a few sinuses cancer) and non-locally advanced, or with a combination of daily radiotherapy with chemotherapy (usually with platinum-based compounds) with neoadjuvant or even curative intents [10, 68]. Even if radio-chemotherapy can achieve complete responses also in cases of locally advanced disease, local and/or metastatic recurrence is inevitable in almost half of the patients [69]. The association with targeted therapies, such as the case of Cetuximab, has shown incremented activity, but still failing in prolonging overall survival.

HNSCC shows an extremely high percentage of mutations in the p53 gene, up to the 70% of cases [24]. Indeed, like in several other cancers [70–73], p53 and its family members seems to be of high relevance. Anyhow, p63, another p53 family member, has been recognized as playing a pathogenetic role in this cancer since its gene locus is often amplified. TP63 gene deregulation is a frequent event in squamous cancers (it can be seen also in esophageal, lung and cervix cancer) since p63 is involved in the maintenance of epithelial homeostasis, especially in those cells in which p53 is somehow inactivated [74]. Being a transcription factor, p63 activity has been described in various circumstances in different cancers, sustaining cell survival, cell renewal and reducing senescence [45]. Moreover, since epithelial tissues require a high metabolic energetic flux due to constant proliferation, p63 has been described in regulating cellular metabolism in normal and pathological conditions such as cancer [40].

Cancer cells have a continuous demand for energy production, therefore their metabolism is reshaped to produce a greater number of Krebs cycle intermediates to refuel the respiratory chain with electrons [75, 76]. Numerous catalytic and anabolic metabolisms, which can provide such intermediates, are altered in cancer. Among them the serine de novo biosynthesis and one carbon metabolism are often deregulated since they can provide Krebs cycle intermediates from glycolysis derived pyruvate (in the usually glycolytic environment of the cancer cell), providing also the hydroxy-methyl groups which are essential for chromatin remodeling (through the synthesis of s-adenosylmethionine), glutathione and purine synthesis, which are also fundamental for cell proliferation [57, 77].

In HNSCC the expression of genes coding for enzymes involved either in the serine de novo biosynthesis and the OCM have been found deregulated at a genomic and transcriptomic level in a cohort of patients form the TCGA. In this group of patients, most of the enzymes of the two metabolisms show altered RNA expression, but the most striking altered expressed gene is PSPH, altered in up to the 11% of patients, most invariably amplified. The alteration of the expression levels of these genes seems to be specific for cancer cells since their expression levels in HNSCC tissues are invariably higher if compared to their normal matching controls.

## Conclusion

P63, like its family members, has already been demonstrated to control specific cell metabolism in various contexts either through its transcriptional activity or through direct transactivation of key enzymes such as hexokinase 2 [40, 42, 49]. In keratinocytes, p63 has a direct interaction with the promoter regions of many of the genes involved in serine de novo biosynthesis and OCM (*PHGDH, PSAT1, PSPH* and *MTHFD2*), which is also true in FaDu cells, a well-established HNSCC cell line model, where p63 shows the same binding to the promoter region of the abovementioned genes. In FaDu cells, the silencing of p63 reduces the expression levels of the genes where its binding on the promoter has been shown and decreases the protein levels of the enzyme PSPH, the last enzyme of the serine biosynthesis.

Since the alteration of p63 in HNSCC is a documented event, the serine de novo biosynthesis and the OCM alteration, which can be under the regulation of p63 itself in HNSCC should be better evaluated, representing a potential therapeutical target since many compounds are rapidly being made available for modulating the activity of the enzymes of these two metabolisms (e.g. PHGDH inhibitors, SHMT2 inhibitors) and the influence of metabolism on sensitivity to therapy has been demonstrated in various tumoral contexts [63, 78–80]. Moreover, apart from therapeutical purposes, the alteration of the genes/enzymes of the two metabolisms, should also be thoroughly evaluated as predictive biomarkers, since a 5-genes signature (*TP63, PHGDH, PSAT1, PSPH*) seems to correlate with HNSCC overall survival and considering the lack of effective predictive tools [81–83].

Altogether our findings show a possible involvement of serine de novo synthesis and OCM in HNSCC pathogenesis, potentially under the control of p63, representing the first step towards the understanding of the metabolic reshape of this heterogeneous group of malignancies.

## Material and methods

### In vitro cell culture and transfection

FaDu cells were seeded, in MEM (Corning, Cat. No. 10-109-CV) enriched of 10% FBS, 2 mM L-glutamine, 10 mM HEPES, 1 mM sodium pyruvate, 100 u/ml penicillin, and 100 mg/mL streptomycin. The cells were cultured in the medium at 37 °C and 5% CO_2_. FaDu cell line was transfected for siRNA-mediated knockdown experiments for 48 and 72 h. The cells (6 × 10^5^ cells) were seeded in 10 mm plates and transfected with specific siRNAs for p63 (Table 1) using Lipofectamine RNAiMAX transfection reagent (Invitrogen).

**Table 1:** siRNA sequences

h-p63-1	5′-GGAUGAACCGCCGUCCAAU-3′
h-p63-2	5′-CAGGUUGGCACUGAAUUCA-3′

### RNA extraction and RT-qPCR analysis

Total RNA was isolated from FaDu cells by using RLT lysis buffer (Qiagen) and it was purified using RNeasy Mini Kit (Qiagen). RNA quantification was performed by NanoDrop spectrophotometer (Thermo Scientific). cDNA was synthesized using SensiFAST™ cDNA synthesis kit (Bioline). The qPCR was performed by GoTaq Real-Time PCR System (Promega) in an Applied Biosystems 7500 Real-Time 15 PCR System (Applied Biosystems). Appropriate qPCR primers (Table 2) were used for quantitative PCR; The housekeeping gene TBP is used as internal control for data normalization. The expression of each gene was defined by the threshold cycle (Ct), and relative expression levels were calculated by using the 2^−ΔΔCt^ method.

**Table 2:** qPCR primers

*hTP63 Fw*	5′-GAAGAAAGGACAGCAGCATTG-3′
*hTP63 Rv*	5′-GGGACTGGTGGACGAGGAG-3′
*hPHGDH Fw*	5′-CACTGAGGCTGTTCCCATT-3′
*hPHGDH Rv*	5′-GTCATCAACGCAGCTGAGAA-3′
*hPSAT1 Fw*	5′-TCATCACGGACAATCACCAC-3′
*hPSAT1 Rv*	5′-GTCCTCAAACTTCCTGTCCAA-3′
*hPSPH Fw*	5′-CATGATTGGAGATGGTGCCA-3′
*hPSPH Rv*	5′-TTATCCTTGACTTGTTGCCTGA-3′
*hMTHFD2 Fw*	5′-CTACTGTGTCTTCTGTGTCAC-3′
*hMTHFD2 Rv*	5′-CTGCATGATATCGGAATGCTC-3′
*hTBP Fw*	5′-TCAAACCCAGAATTTGTTCTCCTTAT-3′
*hTBP Rv*	5′-CCTGAATCCCTTTAGAATAGGGTAG-3′

### Immunoblotting analysis

FaDu cells were lysed with RIPA lysis buffer (50 mM Tris–cl pH 7.4; 150 mM NaCl; 1% NP40; 0.25% Na-deoxycholate; 1 mM AEBSF; and 1 mM DTT). The total protein extracts (20 µg) were separated using SDS polyacrylamide gels and transferred on PVDF membrane. Then, the membranes were incubated overnight at 4 °C with the primary antibody. The membranes were incubated with secondary antibody, later PBS-T washes, for 1 h at room temperature. Immunoblotting signals were captured using Uvitec Alliance Q9 Advanced (PMID: 9531492; PMID: 9794797; PMID: 15698515). The following primary antibodies were implied for western blot experiments: anti-p63 (Cell Signalling, Cat. No. 13109, 1:1000), anti-PHGDH (Sigma, Cat. No. HPA021241, 1:1000), anti-PSAT1 (Abcam ab154055, 1:500), anti-PSPH (Sigma, Cat. No. HPA020376, 1:300), anti-MTHFD2 (Abcam Cat. No. ab151447, 1:1000) and anti-vinculin (Sigma, Cat. No. V9131 1:10000). Uncropped images of the western blots are shown in additional file 1.

### Statistical analysis

Data, which represent three independent experiments, are expressed as means ± SDs. GraphPad Prism 8.0 software (San Diego, CA, USA) was performed for statistical analysis, ordinary one-way analysis of variance (ANOVA) and parametric Student’s t test was applied for comparing different samples. Values of *P* < 0.05 was considered statistically significant.

### Chromatin immunoprecipitation (ChIP) analysis

FaDu cells were cross-linked with 1% formaldehyde (Sigma, Cat. No F1635) for 10 min at room temperature to perform ChIP assay. The cross-linking reaction was blocked by addition of glycine (Sigma, Cat. No G-7126) 125 mM. After washing three times with PBS, chromatin was collected in cell lysis buffer (Pipes 5 mM pH 8.0, KCl 85 mM, NP40 0,5%) for 5 min at 4 °C. Later, chromatin was transferred in nuclei lysis buffer (Tris–Cl 50 mM pH8.0, EDTA 10 mM, SDS1%) and fragmented by sonication. To verify the amount of the chromatin in a range of 250–500 bp, after sonication, it was applied gel electrophoresis. Immunoprecipitation assay was performed overnight using Dynalbeads Protein G (Invitrogen, Cat. No 10004D) and antibodies (5–10 μg) anti-p63 (Cell Signaling, Cat. No. ab13109) or IgG (Invitrogen, Cat. No. 10500C), as control. Immunoprecipitates were washed in IP dilution buffer (SDS 0,01%, Triton X-100 1,1%, EDTA 1,2 mM, Tris–Cl 16,7 mM pH8, NaCl 167 mM) for 3 times, in dialysis buffer (EDTA 2 mM, Tris–Cl 50 mM pH8,0, Sarkosyl 0,2%) for 3 times, in TSE buffer (SDS 0,1%, Triton X-100 1%, Tris 20 mM pH8.1, EDTA 2 mM, NaCl 50 mM) for 3 times, in IP Wash buffer (Tris–Cl 100 mM pH9.0, LiCl 500 mM, NP40 1%, Deoxycholic acid 1%) for 3 times and TE (Tris–Cl pH7.0, EDTA 2 mM) for 1 time. Immunoprecipitation was eluted in elution buffer (NaHCO3 50 mM, SDS 1%) at 65 °C overnight and at least purificated by QIAquick PCR purification kit (Qiagen, Cat. No. 28104). ChIP-qPCR was performed using primers (Table 3) in an Applied Biosystems 7500 Real-Time 15 PCR System (Applied Biosystems, Carlsbad, CA). Real-Time PCR data were normalized to the amount of input chromatin.

**Table 3:** ChIP primers for qPCR

*PHGDH Fw*	5′-AGGGCTAGCTAGGGTCAATAA-3′
*PHGDH Rv*	5′-CCGAGAGACAAAGTAGCAAACA-3′
*PSAT1 Fw*	5′-AGCATTGAAGAAACTGAGCTAGA-3′
*PSAT1 Rv*	5′-GAATAGCGATAAGACCACCAA-3′
*PSPH Fw*	5′-AAGGAGAGACATGAAATAAGTTTGG-3′
*PSPH Rv*	5′-GCCGTTTGCAGCACTTT-3′
*MTHFD2 Fw*	5′-CAGGACAAGAACTCAGGGATT-3′
*MTHFD2 Rv*	5′-GCCTGCCACAGGAGTTG-3′

### Bioinformatics analysis

The Oncoprint profiling and Patients’ overlap analysis of expression alterations of TP63, serine metabolism and OCM enzymes in HNSCC (TCGA firehose legacy) was performed using cBioPortal for Cancer Genomic (http://www.cbioportal.org).

For TP63, serine metabolism and OCM enzymes expression at mRNA level in FaDu and in HaCaT cells two publicly databases available through GEO datasets (https://www.ncbi.nlm.nih.gov/gds) were analyzed (GSE88833 and GSE88832 respectively). ChIP sequencing publicly data (GSE126390, GSE33571, GSE56674, GSE60814, GSE67382, GSE59827) was visualized using Genome Browser (https://genome.ucsc.edu/). The correlation analysis between p63 (protein) and *PHGDH*, *PSAT1*, *PSPH* and MTHFD2 (mRNA) in HNSCC (TCGA firehose legacy) was performed using cBioPortal for Cancer Genomic (http://www.cbioportal.org).

### Supplementary Information

**Additional file 1: Fig S1.** Serine and OCM enzymes are differentially expressed in single-cell RNAseq of HNSCC and leukoplakia samples. (a) Average expression of serine and one carbon metabolism enzymes in single-cell RNA sequencing of normal and HNSCC samples (GSE181919); NL: non lesional samples; LP: Leukoplakia samples; LN: Lymph nodes + samples; CA: primary cancer samples (b) *PHGDH, PSAT1, PSPH* and *MTHFD2* expression in all cell types. (c) *PHGDH, PSAT1, PSPH* and *MTHFD2* expression in leukoplakia samples cell types. **Fig S2.** Serine and OCM enzymes are differentially expressed in single-cell RNAseq of normal and HNSCC samples. (a) *PHGDH, PSAT1, PSPH* and *MTHFD2* expression in single-cell RNA seq of normal cell types (GSE181919) (b) *PHGDH, PSAT1, PSPH* and *MTHFD2* expression in single-cell RNA seq of primary tumor samples cell types. (GSE181919) (c) *PHGDH, PSAT1, PSPH* and *MTHFD2* expression in single-cell RNA seq of metastatic samples cell types, (GSE181919). **Fig. S3.** p63 controls PHGDH, PSAT1 and MTHFD2 expression in FaDu and HaCaT cells. (a) correlation analysis of the mRNA expression of p63 (protein) and *PHGDH*, *PSAT1*, *PSPH* and *MTHFD2* (mRNA) in HNSCC (TCGA firehose legacy). (b) Expression analysis of *PHGDH*, *PSAT1, PSPH* and *MTHFD2* in a publicly available dataset of FaDu cells (ctr vs sh-p63) (GSE88833). (c) Analysis of *PHGDH, PSAT1*, *PSPH* and *MTHFD2* mRNA expression in a publicly available dataset of HaCaT cells (ctr vs sh-p63) (GSE88832); the p-value was obtained using ordinary one-way analysis of variance (ANOVA). Values were considered significant when the p-value < 0.05 (*n.s*. = not significant). **Fig. S4.** Western blots uncropped images. Corresponding panels used in the main figures are indicated.

## Data Availability

For Serine metabolism and OCM enzymes expression at mRNA level in HNSCC samples a publicly datasets available through GEO datasets (https://www.ncbi.nlm.nih.gov/gds) was analyzed (GSE12452). The expression of serine and OCM metabolism enzymes in NL: non lesional samples; LP: Leukoplakia samples; LN: Lymph nodes + samples; CA: primary cancer samples of HNSCC was analyzed using through Single Cell RNA sequencing publicly datasets GEO datasets (GSE181919). For p63 binding on serine and OCM enzymes promoter regions different Chromatin Immunoprecipitation sequencings (ChIP seq) (GSE126390, GSE33571, GSE56674, GSE60814, GSE67382, GSE59827) were visualized and analyzed using UCSC genome Browser (https://genome.ucsc.edu/).

## Abbreviations

HNSCC: Head and neck squamous cell carcinoma
HK2: Hexokinase 2
PHGDH: Phosphoglycerate dehydrogenase
PSAT1: Phosphoserine aminotransferase 1
PSPH: Phosphoserine phosphatase
SHMT1: Serine hydroxymethyltransferases 1
SHMT2: Serine hydroxymethyltransferases 2
THF: Tetrahydrofolate
Me-THF: Methylenetetrahydrofolate
GSH: Glutathione
OCM: One carbon metabolism
MTHFD2: Methylenetetrahydrofolate dehydrogenase (NADP+ dependent) 2
