# Supplementary material for: p63 orchestrates serine and one carbon metabolism enzymes expression in head and neck cancer
Source: Biol Direct. 2023 Nov 9;18:73. doi: 10.1186/s13062-023-00426-1 (PMC10636826; doi:10.1186/s13062-023-00426-1)

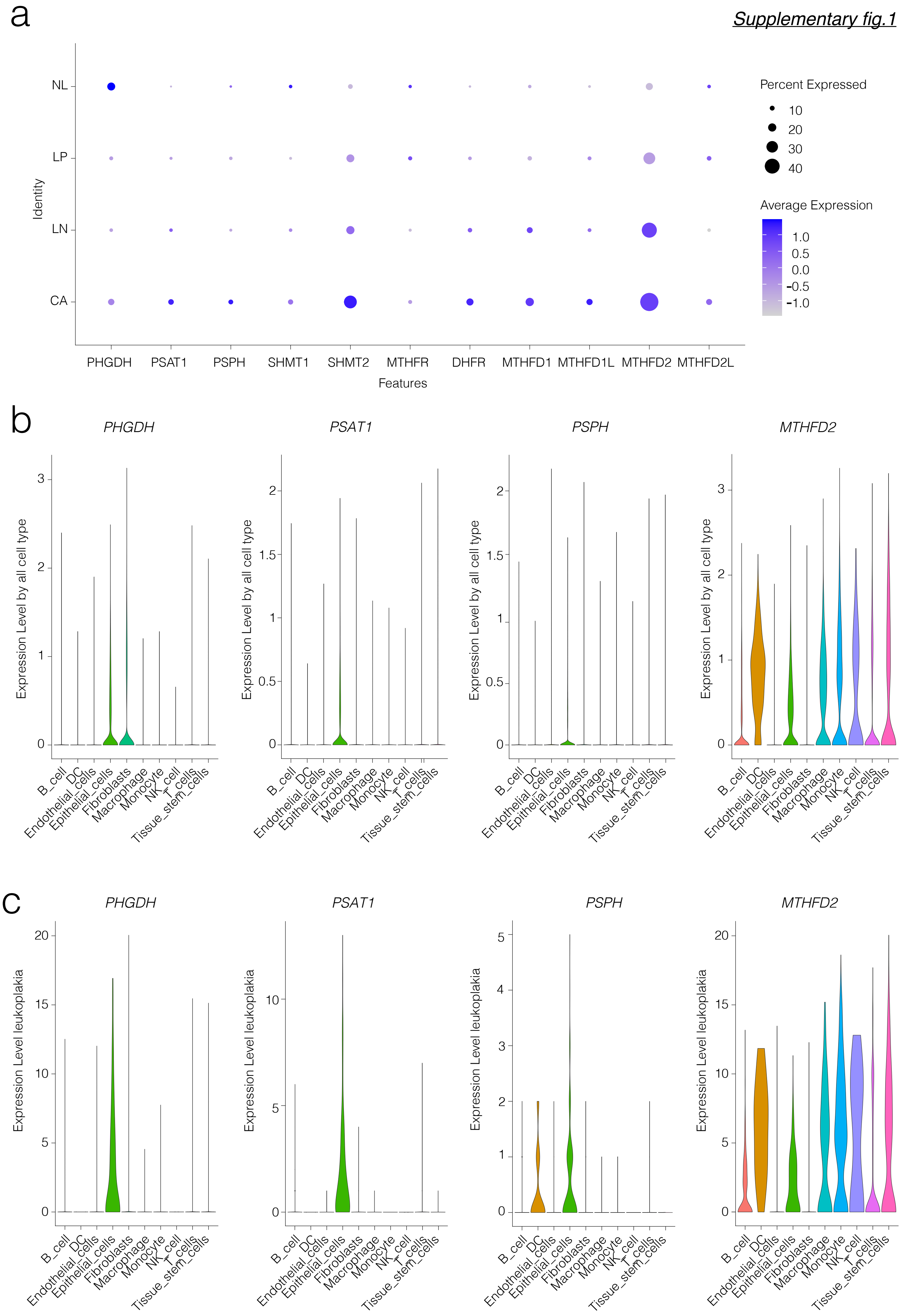
a
NL
LP
LN
CA
Identity
PHGDH
PSAT1
PSPH
SHMT1
SHMT2
MTHFR
DHFR
MTHFD1
MTHFD1L
MTHFD2
MTHFD2L
Features
Percent Expressed
10
20
30
40
Average Expression
1.0
0.5
0.0
−0.5
−1.0
b
PHGDH
PSAT1
PSPH
MTHFD2
Expression Level by all cell type
B_cell
DC
Endothelial_cells
Epithelial_cells
Fibroblasts
Macrophage
Monocyte
NK_cell
T_cells
Tissue_stem_cells
c
PHGDH
PSAT1
PSPH
MTHFD2
Expression Level leukoplakia

Supplementary fig.2

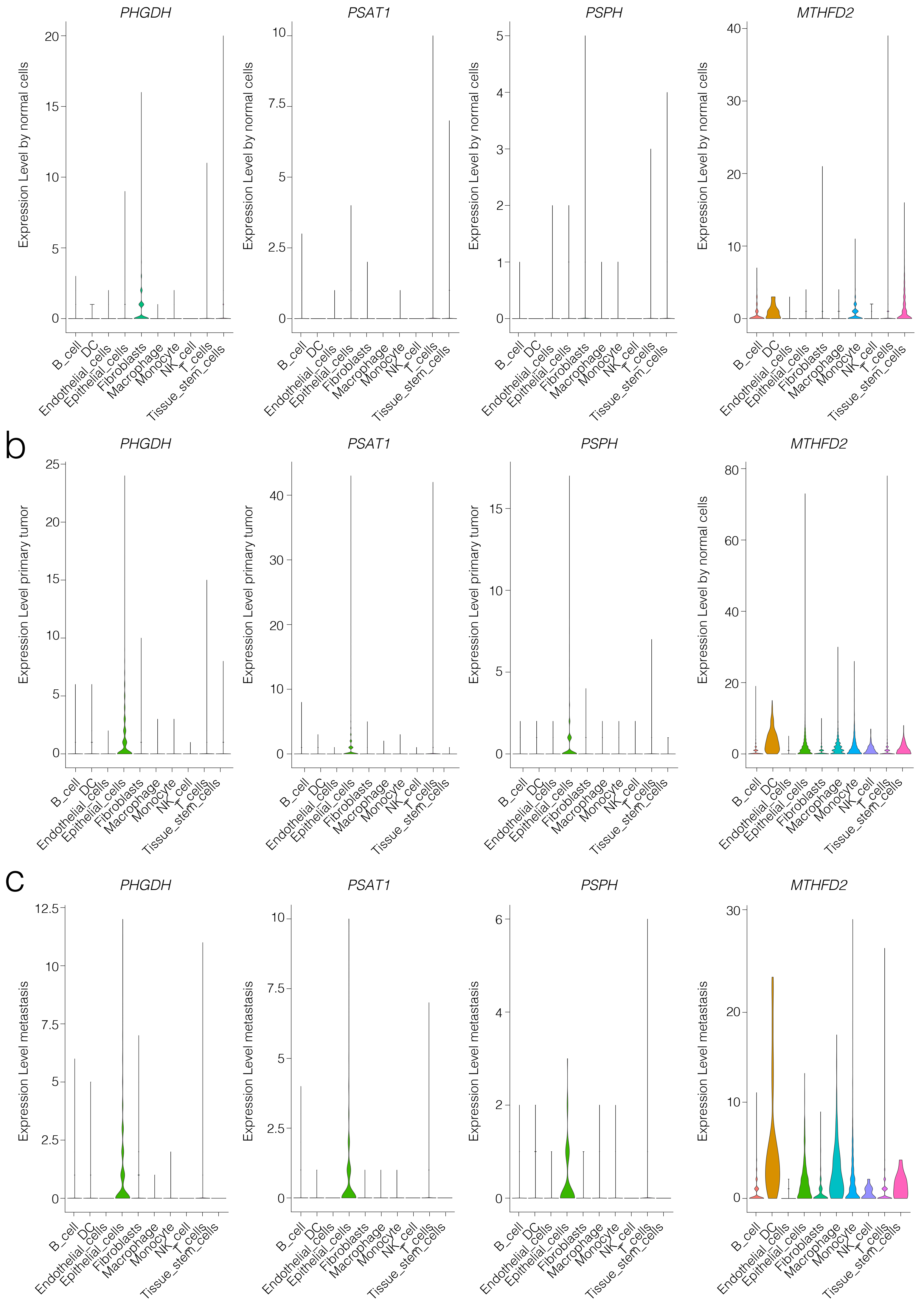

Supplementary fig.3

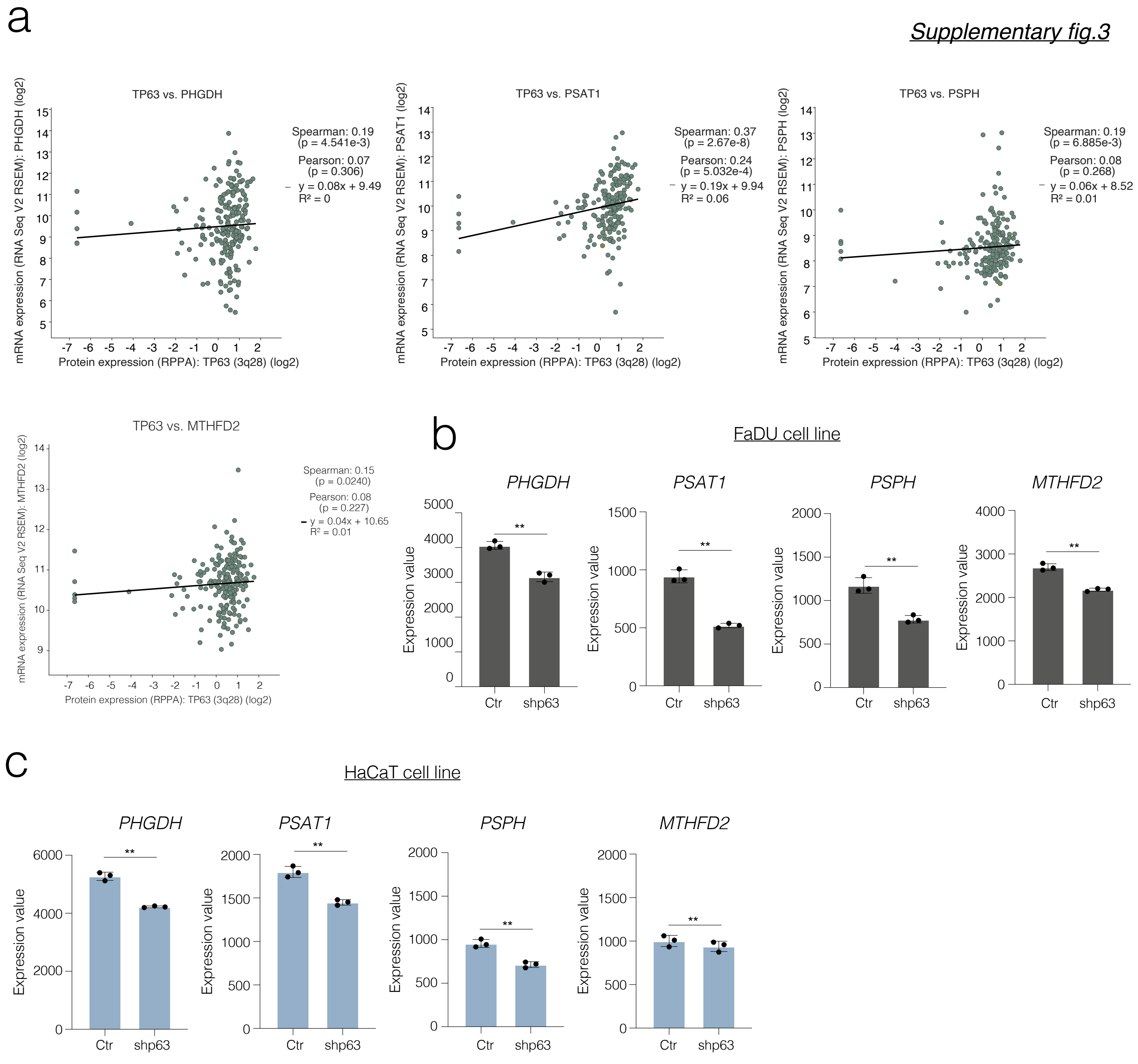

UVITEC photo documenter

Fig.3 panel C

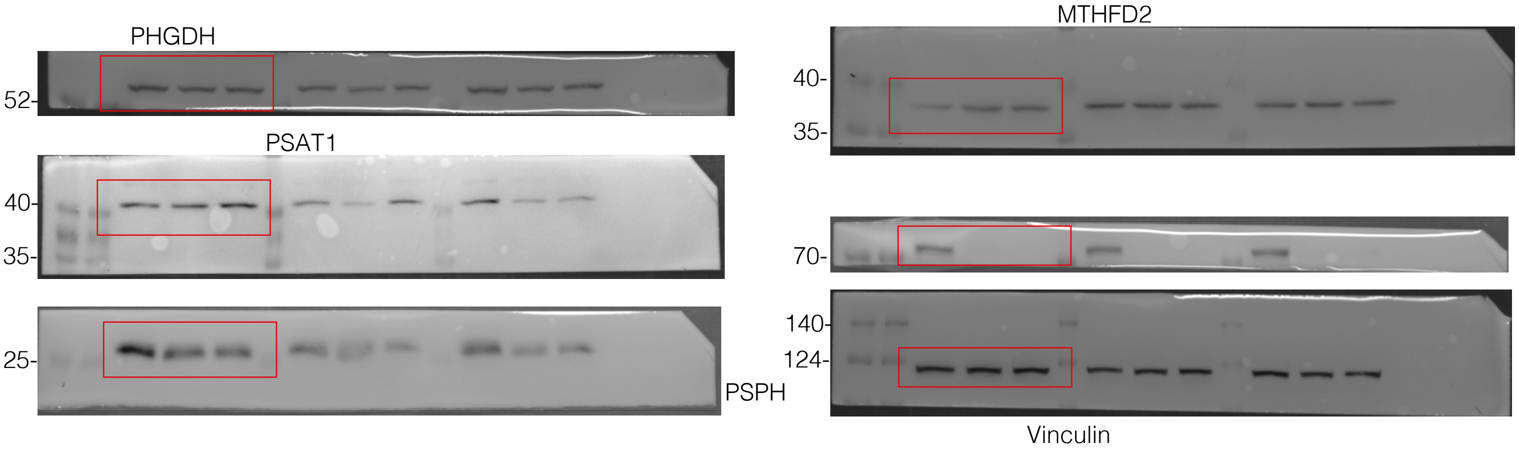

Supplement: Supplementary file 1 — Additional file 1: Fig S1. Serine and OCM enzymes are differentially expressed in single-cell RNAseq of HNSCC and leukoplakia samples. (a) Average expression of serine and one carbon metabolism enzymes in single-cell RNA sequencing of normal and HNSCC samples (GSE181919); NL: non lesional samples; LP: Leukoplakia samples; LN: Lymph nodes + samples; CA: primary cancer samples (b) PHGDH, PSAT1, PSPH and MTHFD2 expression in all cell types. (c) PHGDH, PSAT1, PSPH and MTHFD2 expression in leukoplakia samples cell types. Fig S2. Serine and OCM enzymes are differentially expressed in single-cell RNAseq of normal and HNSCC samples. (a) PHGDH, PSAT1, PSPH and MTHFD2 expression in single-cell RNA seq of normal cell types (GSE181919) (b) PHGDH, PSAT1, PSPH and MTHFD2 expression in single-cell RNA seq of primary tumor samples cell types. (GSE181919) (c) PHGDH, PSAT1, PSPH and MTHFD2 expression in single-cell RNA seq of metastatic samples cell types, (GSE181919). Fig. S3. p63 controls PHGDH, PSAT1 and MTHFD2 expression in FaDu and HaCaT cells. (a) correlation analysis of the mRNA expression of p63 (protein) and PHGDH, PSAT1, PSPH and MTHFD2 (mRNA) in HNSCC (TCGA firehose legacy). (b) Expression analysis of PHGDH, PSAT1, PSPH and MTHFD2 in a publicly available dataset of FaDu cells (ctr vs sh-p63) (GSE88833). (c) Analysis of PHGDH, PSAT1, PSPH and MTHFD2 mRNA expression in a publicly available dataset of HaCaT cells (ctr vs sh-p63) (GSE88832); the p-value was obtained using ordinary one-way analysis of variance (ANOVA). Values were considered significant when the p-value < 0.05 (n.s. = not significant). Fig. S4. Western blots uncropped images. Corresponding panels used in the main figures are indicated. [file 13062_2023_426_MOESM1_ESM.pdf]
